# Current Practices in Antibiotic Prophylaxis for Transoral Endoscopic Thyroid and Parathyroid Surgery: A Comparative Study

**DOI:** 10.3390/medicina61050939

**Published:** 2025-05-21

**Authors:** Mehmet Ilker Turan, Senay Ozturk Durmaz, Mehmet Celik, Nedim Akgul

**Affiliations:** 1 Department of General Surgery, Kepez State Hospital, 07320 Antalya, Turkey; 2 Department of Infectious Diseases and Clinical Microbiology, Kepez State Hospital, 07320 Antalya, Turkey; drsenay70@gmail.com; 3 Department of Endocrinology and Metabolism, Medical Faculty, Trakya University, 22000 Edirne, Turkey; drmehmetcelik@hotmail.com; 4 Department of General Surgery, Antalya Training and Research Hospital, University of Health Sciences, 07070 Antalya, Turkey; nedimakgul@yahoo.com

**Keywords:** transoral endoscopic thyroidectomy, antibiotic prophylaxis, surgical site infections, scar-free thyroid surgery, learning curve

## Abstract

*Background and Objectives:* The transoral endoscopic thyroidectomy-vestibular approach (TOETVA) and parathyroidectomy-vestibular approach (TOEPVA) are scar-free alternatives to conventional surgery but are classified as clean-contaminated due to the oral incision, raising concerns about surgical site infections (SSIs). This study evaluates whether perioperative antibiotic prophylaxis (pABX) alone is sufficient compared to extended antibiotic prophylaxis (eABX) in preventing SSIs in TOET/PVA, particularly considering the surgical learning curve. *Materials and Methods:* A retrospective study analyzed 162 patients undergoing TOET/PVA at a single center from January 2018 to June 2024. Patients were divided into two groups: 82 received eABX (intravenous cefazolin preoperatively plus 7 days of oral amoxicillin/clavulanate), and 80 received pABX alone (intravenous cefazolin). The inclusion criteria included complete postoperative hemogram and C-reactive protein (CRP) records; exclusions comprised other surgical approaches or missing data. Outcomes included postoperative white blood cell (WBC) count, CRP levels, and complications (seroma, cellulitis, and flap perforation), defined using Centers for Disease Control and Prevention (CDC) guidelines. The statistical analysis comprised *t*-tests, chi-square tests, and logistic regression, adjusting for confounders like age and sex. *Results:* The postoperative WBC and CRP levels were significantly higher in the pABX group (*p* = 0.001), but all values remained within the laboratory normal limits. Complications were observed in 14 patients: seroma in 11, cellulitis in 2, and flap perforation in 1. Complications occurred more frequently in the eABX group but without statistical significance (*p* = 0.103). The duration of surgery was longer in the eABX group (117.93 ± 52.35 vs. 72.44 ± 22.54 min, *p* = 0.001) and was an independent predictor of complications (OR = 1.018, 95% CI: 1.006–1.031, *p* = 0.004). *Conclusions:* Perioperative antibiotic prophylaxis alone does not increase the risk of SSIs compared to extended prophylaxis in TOETVA. However, eABX may be prudent during the learning curve due to longer operative times and higher complication risks. Future prospective, randomized trials are needed to standardize prophylaxis regimens.

## 1. Introduction

Conventional open thyroidectomy has been performed effectively and reliably, until today. This technique, performed via a transcervical incision, is considered the gold standard [1]. Conventional open thyroidectomy is classified as “clean surgery” in terms of the risk of surgical site infection (SSI) [2]. Thus, routine antibiotic prophylaxis is not recommended [3].

Conventional open thyroidectomy leaves a visible scar on the neck. Scar perception varies by cultural factors, ethnicity, age, and gender, often impacting patients’ quality of life [4,5,6,7]. To address this, remote-access techniques like the transoral endoscopic thyroidectomy-vestibular approach (TOETVA) and parathyroidectomy-vestibular approach (TOEPVA) have emerged, offering scar-free outcomes [8,9,10]. Like every new surgical technique, TOETVA has its own set of complications such as mental nerve injury and carbon dioxide embolism [10]. Unlike conventional thyroidectomy, classified as clean surgery with low SSI rates (0.3–0.6%) and no routine antibiotic prophylaxis recommended [2,3], TOET/PVA is clean-contaminated due to the oral incision, potentially increasing the SSI risk [11]. Thus, the initial protocols for TOET/PVA recommended perioperative antibiotic prophylaxis (pABX) plus postoperative chlorhexidine gargles and 3–7 days of oral antibiotics (extended antibiotic prophylaxis, eABX) [12]. However, recent studies suggest that eABX may be unnecessary, citing low infection rates with pABX alone [13,14,15]. Therefore, there is no standard approach in the literature for routine antibiotic prophylaxis in TOETVA.

The absence of standardized guidelines for antibiotic prophylaxis in TOET/PVA, coupled with the surgical learning curve’s impact on operative time and complications, underscores the need to evaluate optimal strategies. This study aimed to compare the efficacy and safety of perioperative antibiotic prophylaxis alone versus postoperative extended oral antibiotic prophylaxis regimens in patients undergoing transoral endoscopic thyroidectomy and/or parathyroidectomy (TOET/PVA).

## 2. Materials and Methods

### 2.1. Study Design

This retrospective study reviewed medical records of 947 patients who underwent thyroidectomy and/or parathyroidectomy at Antalya Kepez State Hospital between January 2018 and June 2024.

Exclusion criteria:

1. Patients undergoing open thyroidectomy/parathyroidectomy (*n* = 746).
2. Patients undergoing endoscopic thyroidectomy via the bilateral axillo-breast approach (BABA) (*n* = 24).
3. Patients lacking postoperative hemogram or C-reactive protein (CRP) records (*n* = 15).

The final cohort comprised 162 patients undergoing TOET/PVA, divided into two groups based on antibiotic prophylaxis:

1. **pABX group (*n* = 80):** Received perioperative intravenous cefazolin (1 g for BMI < 30, 2 g for BMI ≥ 30) 30–60 min preoperatively.
2. **eABX group (*n* = 82):** Received pABX plus 7 days of oral amoxicillin/clavulanate (875 mg twice daily) postoperatively.

**Figure 1:**
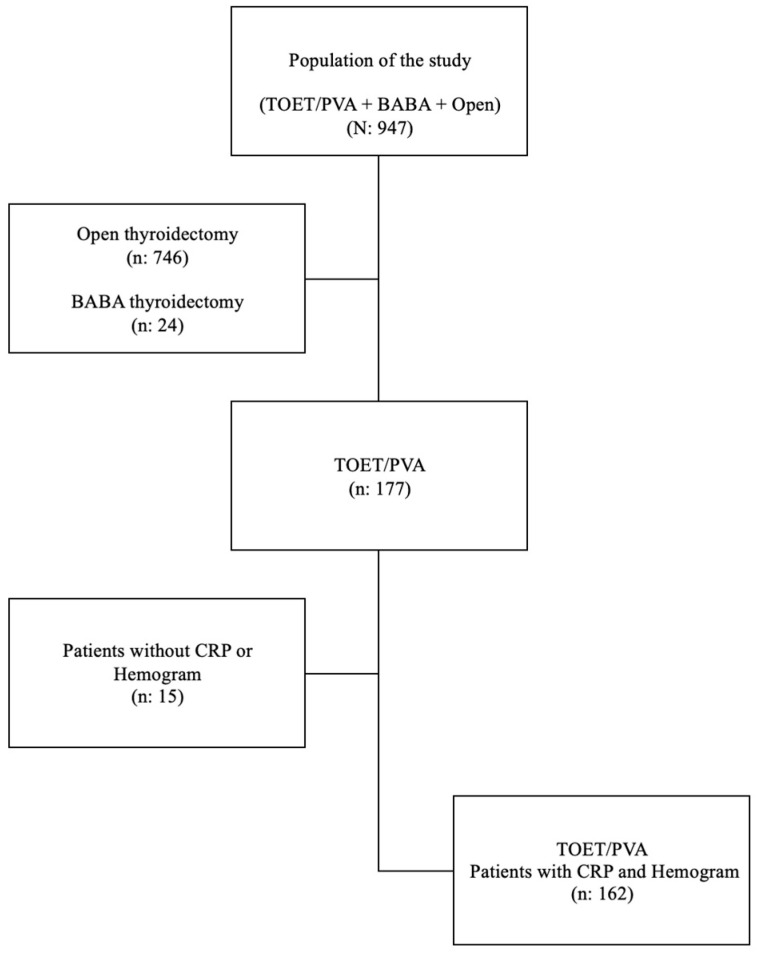
Flow chart of the study. Abbreviations: TOET/PVA: transoral endoscopic thyroidectomy and/or parathyroidectomy-vestibular approach, BABA: bilateral axillo-breast approach, CRP: C-reactive protein.

The patient selection scheme is shown in Figure 1.

Demographic data, such as age, sex, and body mass index (BMI), as well as operative data, including the type and dose of intravenous antibiotics administered as perioperative prophylaxis and the duration of surgery, were extracted from electronic medical records. In addition, the white blood cell (WBC) and CRP levels on postoperative day 1, were noted. Complications, including seroma, cellulitis, and flap perforation, were defined as per Centers for Disease Control and Prevention (CDC) guidelines for SSIs [3]. Seroma was diagnosed via ultrasound, cellulitis by clinical examination (erythema, warmth), and flap perforation by direct visualization.

In the group that received perioperative antibiotic prophylaxis alone (pABX), 1 g of cefazolin was administered intravenously to patients with a body mass index of less than 30 and 2 g to those with a body mass index of more than 30, 30–60 min before surgery. In the extended antibiotic prophylaxis group (eABX), 875 mg amoxicillin/clavulanate was given orally twice a day for 7 days postoperatively, in addition to cefazolin given for perioperative antibiotic prophylaxis. In our clinic, extended oral antibiotic prophylaxis was administered postoperatively, in addition to perioperative prophylaxis, for surgeries conducted till June 2021. As of June 2021, perioperative antibiotic prophylaxis alone has been implemented, reflecting evolving institutional practices and surgeon discretion. All patients were operated on by the same surgeon (M.I.T). Approval for the study was obtained from the Clinical Research Ethics Committee of the University of Health Sciences, Antalya Training and Research Hospital (Ethics Committee approval number: 2024-386).

### 2.2. Surgical Technique

**Figure 2:**
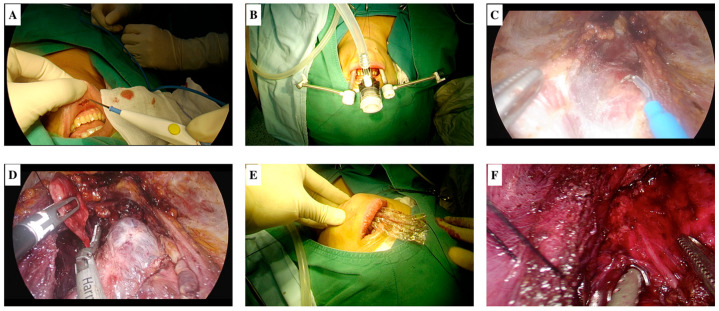
Operative steps. (**A**) Oral vestibule incision; (**B**) external view of all trocars; (**C**) endoscopic view of opening of the midline; (**D**) endoscopic view of resection of left lower parathyroid; (**E**) removal of the specimen via endo-bag; (**F**) endoscopic appearance of the recurrent laryngeal nerve after resection.

All patients were consulted by a dentist before surgery, and chlorhexidine oral gargle was administered 3 times a day for 3 days preoperatively. Thirty to sixty minutes before surgery, patients with a BMI below 30 were given intravenous cefazolin 1 g (cefazolin sodium); patients with a BMI above 30 were given intravenous cefazolin 2 g prophylactically. The patient was placed in the supine position and intubated orotracheally with a neuromonitoring-compatible intubation tube. The oral cavity was washed with chlorhexidine solution. Next, a 1.5–2 cm transverse incision was made on the lower lip, just above the frenulum. From this incision, dissection was performed with the help of electrocautery, just above the periosteum towards the tip of the chin. Then, hydrodissection was performed along the line extending from the tip of the chin to the sternal notch, using a solution prepared by adding 1 mg adrenaline to 500 cc of isotonic. Subsequently, subplatysmal tunnels were created from this space with the help of a dilator. A 10 mm trocar was placed through the incision, and carbon dioxide insufflation was performed through this trocar. A 2/0 silk suture was placed approximately 1 cm distal to the tip of the trocar for skin traction. Subsequently, two 5 mm trocars were placed through the incisions made just below the lower lip and laterally. By combining the subplatysmal tunnels with the help of energy-based devices, dissection was performed up to the sternal notch at the inferior border and up to the medial aspect of the sternocleidomastoid (SCM) muscle at the lateral borders. The strap muscles were separated from the midline, and the thyroidectomy/parathyroidectomy stage was started. After the resection was completed, the specimen was removed with the help of an endo-bag and the incisions were closed continuously with 3/0 absorbable sutures. The operative steps are shown in Figure 2. Postoperatively, patients were discharged with the recommendation to use chlorhexidine oral gargle 3 times a day for 1 week and/or extended postoperative oral antibiotic prophylaxis.

### 2.3. Statistical Analysis

The data obtained were evaluated statistically using the SPSS (Statistical Package for the Social Sciences Version 22.0; SPSS Inc., Chicago, IL, USA) software. Normality was assessed with the Kolmogorov–Smirnov test. Continuous variables (e.g., WBC, CRP) were presented as mean ± standard deviation and compared using independent samples *t*-tests. Categorical variables (e.g., complication rates) were reported as frequencies (%) and analyzed with chi-square, Fisher’s exact, or Fisher–Freeman–Halton tests. Logistic regression models assessed the predictors of complications, with complication occurrence (yes/no) as the dependent variable. Models adjusted for age, sex, and surgery duration; confounders like diabetes and smoking were evaluated but not included due to low prevalence (<5%). Missing data were minimal (<2%) and handled via listwise deletion. Effect sizes were reported as odds ratios (ORs) with 95% confidence intervals (CIs). Model fit was evaluated using Hosmer–Lemeshow tests and residual analyses. A *p*-value < 0.05 indicated statistical significance.

## 3. Results

The comparative descriptive data are presented in Table 1. The postoperative WBC and postoperative CRP levels showed a statistically significant increase in the group that was only given perioperative antibiotic prophylaxis (*p* = 0.001). However, the increases were limited to laboratory normal ranges. No statistically significant difference was detected in the other comparative analyses.

The operative data and outcome variables are summarized in Table 2. The length of hospital stay and duration of surgery in the patients who received eABX were found to be significantly higher than those in the group that received pABX alone (*p* = 0.001). Flap perforation was observed in one patient, cellulitis in two patients, and seroma in seven patients in the group receiving eABX; seroma was observed in four patients in the group receiving pABX alone. The eABX group had a higher complication rate (12.2% vs. 5.0%), but the difference was not significant (*p* = 0.103, 95% CI: 0.792–8.792). Ultrasound-guided aspiration was performed on the patients who developed seroma. The flap perforation was primarily repaired. The seromas resolved within 7–14 days post-aspiration, cellulitis within 7–10 days post-antibiotics, and flap perforation after primary repair (day 3).

**Figure 3:**
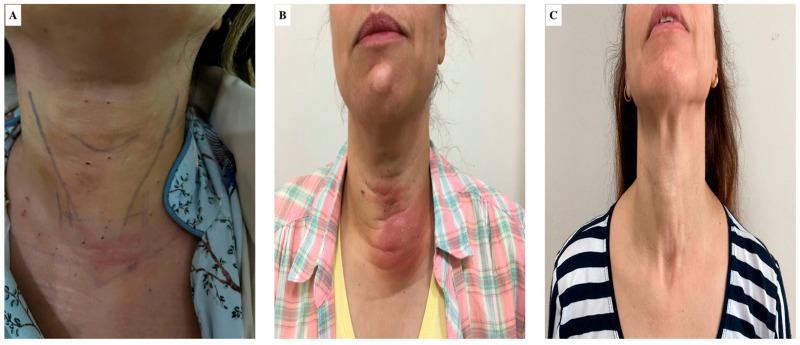
Appearance of the neck of a patient with postoperative cellulite. (**A**) postoperative day 1; (**B**) postoperative day 7; (**C**) postoperative day 30).

The patients who developed cellulitis were followed with oral antibiotic treatment and no surgical procedure was performed (Figure 3). No statistically significant difference was observed between the two groups in terms of the frequency of complications (*p* = 0.103).

Any complication development status (*n*: 14) was accepted as the dependent variable and logistic regression modeling was created in order to show independent predictors. The results are presented in Table 3. In the first model, no statistically significant relationship was found between eABX and the development of complications. In the second modeling, when eABX and the duration of surgery were added together to the analysis, the duration of surgery was found to be an independent predictor (*p* = 0.004, OR = 1.018). When the same model was adjusted for age and sex, the statistical relationship between the duration of surgery and complication development persisted.

## 4. Discussion

This study demonstrates that perioperative antibiotic prophylaxis (pABX) alone appears comparable to extended antibiotic prophylaxis (eABX) in preventing surgical site infections (SSIs) in patients undergoing transoral endoscopic thyroidectomy and/or parathyroidectomy (TOET/PVA), particularly when performed by experienced surgeons. Although the postoperative white blood cell (WBC) and C-reactive protein (CRP) levels were statistically lower in the eABX group, all values remained within laboratory normal limits, suggesting no clinically significant advantage of eABX. Minor complications, such as seroma, flap perforation, and cellulitis, which may predispose to infections, were more frequent in the eABX group, likely due to surgeries performed during the learning curve period. These findings align with the evolving understanding of antibiotic prophylaxis in TOET/PVA and highlight the influence of surgical experience on outcomes.

The results are consistent with several studies suggesting that pABX alone may suffice in TOET/PVA. For instance, Yi et al. [11] reported no benefit of eABX in a randomized trial of 50 thyroid cancer patients undergoing TOETVA, noting potential harm from antibiotic side effects. Similarly, Karakas et al. [14] found low SSI rates without eABX in 113 patients, though their study included heterogeneous surgical techniques (e.g., retroauricular or submental incisions in 36% of cases), limiting direct comparability. Lee et al. [15] further supported this by reporting no need for prophylactic antibiotics in a small cohort of 20 TOET/PVA patients, though bacterial growth in postoperative cultures indicated a potential infection risk. In contrast, our study, with a larger sample size (*n* = 162), found no significant difference in the complication rates between the pABX and eABX groups (*p* = 0.103), suggesting that pABX is likely sufficient for experienced surgeons. However, discrepancies exist in the literature. For example, Yi et al. [11] suggested that eABX might be harmful due to side effects, whereas our study observed no such adverse effects, possibly due to differences in patient populations (e.g., malignancy in all of Yi et al.’s patients vs. mixed indications in ours). Additionally, Zhang et al. [16] highlighted bacterial colonization on endoscopic instruments, which increases with longer operative times, supporting our finding that the duration of surgery is an independent predictor of complications (OR = 1.018, *p* = 0.004). These comparisons underscore a growing consensus that pABX may be adequate for TOET/PVA, but differences in study design, surgical technique, and patient cohorts necessitate cautious interpretation.

The variability in antibiotic prophylaxis practices for TOET/PVA reflects broader inconsistencies in surgical antimicrobial prophylaxis. Studies on conventional thyroidectomy, classified as clean surgery with low SSI rates (0.3–0.6%) [2,17], show that antibiotic prophylaxis is often used despite guideline recommendations against it, with usage rates ranging from 8.8% to 58.3% among surgeons [18,19]. In TOET/PVA, classified as clean-contaminated due to the oral incision, the lack of standardized guidelines leads to similar variability. Our findings suggest that this variability may not significantly impact SSI rates when pABX is used, but the higher complication rates during the learning curve (e.g., 12.2% in the eABX group vs. 5.0% in the pABX group) indicate that eABX may be prudent for less experienced surgeons. Clinically, this variability suggests a need for tailored prophylaxis strategies. For instance, institutions with surgeons early in their TOET/PVA learning curve (typically 40–50 cases [20,21,22,23]) may opt for eABX to mitigate risks associated with longer operative times and suboptimal technique. Conversely, experienced surgeons could safely rely on pABX, reducing unnecessary antibiotic exposure. These findings advocate for the development of evidence-based guidelines that account for surgeon experience and patient-specific factors, such as comorbidities or malignancy, to standardize TOET/PVA prophylaxis protocols.

The choice of antibiotic prophylaxis must balance the prevention of SSIs with the risks of antibiotic overuse. Unnecessary or prolonged antibiotic use can lead to antibiotic resistance, a growing global concern, and complications such as *Clostridium difficile* infection, which carries significant morbidity [3]. In TOET/PVA, the clean-contaminated nature of the surgery justifies prophylaxis, but our study suggests that extending antibiotics beyond the perioperative period offers no clear benefit in experienced hands. The absence of significant differences in the SSI rates between pABX and eABX groups supports minimizing antibiotic use to reduce these risks. However, during the learning curve, when operative times are longer (117.93 ± 52.35 vs. 72.44 ± 22.54 min, *p* = 0.001) and complications are more frequent, eABX may provide a safety net against potential infections, as suggested by the original TOET/PVA protocol [12]. This is particularly relevant given that longer operative times are associated with increased bacterial colonization on endoscopic instruments [22] and are an independent risk factor for complications [24,25].

Given the non-randomized design and the confounding influence of the learning curve in our study, claims of pABX non-inferiority to eABX should be interpreted cautiously. The temporal division of groups (eABX until June 2021, pABX thereafter) introduces potential selection bias, and the majority of eABX patients underwent surgery during the learning curve, which may inflate complication rates. To address these limitations, future research should include prospective randomized controlled trials to definitively compare pABX and eABX in TOET/PVA. Additionally, stratified studies based on surgical experience (e.g., <50 vs. ≥50 cases) and patient risk factors (e.g., diabetes, malignancy) could clarify the optimal prophylaxis regimen for specific contexts. Such studies should also evaluate long-term outcomes, including late-onset complications and antibiotic-related adverse effects, to provide a comprehensive risk–benefit profile.

This study’s strengths include its large sample size (*n* = 162), the largest reported in the TOET/PVA antibiotic prophylaxis literature, and surgical standardization by a single surgeon. However, limitations include the retrospective design, short-term follow-up, and learning curve bias in the eABX group. These factors highlight the need for further research to establish evidence-based prophylaxis guidelines for TOET/PVA, balancing infection prevention with the risks of antibiotic overuse.

## 5. Conclusions

In conclusion, perioperative antibiotic prophylaxis alone appears sufficient to prevent SSIs in TOET/PVA compared to extended prophylaxis, particularly with experienced surgeons. However, extended prophylaxis may reduce risks during the learning curve, when operative times and complications are higher. Given the study’s limitations, including its retrospective design and learning curve bias, prospective randomized trials are essential to establish evidence-based guidelines. Tailoring prophylaxis to surgeon experience and patient factors could optimize outcomes while minimizing antibiotic overuse.

## Figures and Tables

**Table 1:** Comparative descriptive data of study population according to prophylaxis status.

		Prophylaxis Situation		*p* Value
		pABX Alone	eABX	
**Age (years)**		39.03 ± 11.02	40.01 ± 9.60	0.544 *
**Sex**	*Female*	76 (95.0%)	79 (96.3%)	0.675 **
	*Male*	4 (5.0%)	3 (3.7%)	
**BMI (kg/m^2^)**		26.33 ± 5.47	26.84 ± 4.55	0.525 *
**Parathormone (pg/mL)**	*Preoperative*	86.96 ± 74.87	80.12 ± 83.97	0.585 *
	*Postoperative*	34.99 ± 20.53	37.18 ± 18.00	0.469 *
**Calcium (mg/dL)**	*Preoperative*	9.89 ± 1.52	9.68 ± 1.07	0.314 *
	*Postoperative*	8.67 ± 0.61	8.50 ± 0.55	0.062 *
**Albumin (g/L)**	*Preoperative*	4.62 ± 0.28	4.30 ± 0.41	0.001 *
	*Postoperative*	4.51 ± 0.28	4.50 ± 3.88	0.984 *
**WBC (10^3^/µL)**	*Preoperative*	5.56 ± 0.73	6.19 ± 1.02	0.135 *
	*Postoperative*	8.37 ± 0.78	7.04 ± 1.03	0.001 *
**CRP (0–5 mg/L)**	*Preoperative*	0.26 ± 0.42	0.28 ± 0.47	0.816 *
	*Postoperative*	3.73 ± 1.07	1.66 ± 0.67	0.001 *
**Vitamin D (µg/L)**	*Preoperative*	28.18 ± 8.38	29.97 ± 6.68	0.001 *
**Free T3 (ng/L)**	*Preoperative*	3.41 ± 0.46	3.18 ± 0.45	0.002 *
**Free T4 (ng/dL)**	*Preoperative*	1.18 ± 0.22	1.22 ± 0.33	0.529 *
**TSH (mIU/1)**	*Preoperative*	2.34 ± 1.48	2.10 ± 1.44	0.307 *

* Independent samples *t*-test, ** chi-square test. Abbreviations: BMI: body mass index; WBC: white blood cell; CRP: C-reactive protein; TSH: thyroid-stimulating hormone; pABX: perioperative antibiotic prophylaxis; eABX: extended antibiotic prophylaxis.

**Table 2:** Operative outcomes and postoperative complications.

		Prophylaxis Situation		*p* Value
		pABX Alone	eABX	
**Surgical procedure**	Right lobectomy	12 (15.0%)	13 (15.9%)	0.103 *
	Left lower-PTX	9 (11.3%)	1 (1.2%)	
	Left upper-PTX	1 (1.3%)	0	
	Right lobectomy + Right upper-PTX	0 (0.0%)	1 (1.2%)	
	Right lobectomy + Right lower-PTX	1 (1.3%)	2 (2.4%)	
	Left lobectomy	20 (25.0%)	18 (22.0%)	
	Left lobectomy + Left lower-PTX	0 (0.0%)	3 (3.7%)	
	Total thyroidectomy	34 (42.5%)	40 (48.8%)	
	Total thyroidectomy + Right lower-PTX	0 (0.0%)	1 (1.2%)	
	Total thyroidectomy + Left lower-PTX	1 (1.3%)	1 (1.2%)	
	Left lobectomy + Right upper-PTX	0 (0.0%)	1 (1.2%)	
	Right lower-PTX	1 (1.3%)	1 (1.2%)	
**Duration of surgery (minutes)**		72.44 ± 22.54	117.93 ± 52.35	0.001 **
**Duration of hospital stay (hours)**		24.90 ± 4.58	31.61 ± 11.24	0.001 **
**Postoperative complications**	Negative	76 (95.0%)	72 (87.8%)	0.103 ***
	Positive	4 (5.0%)	10 (12.2%)	
**Complication type**	Flap perforation	0 (0.0%)	1 (10.0%)	0.466 **
	Cellulite	0 (0.0%)	2 (20.0%)	
	Seroma	4 (100.0%)	7 (70.0%)	

* Fisher–Freeman–Halton test, ** independent samples *t*-test, *** chi-square test. Abbreviations: PTX: parathyroidectomy; pABX: perioperative antibiotic prophylaxis; eABX: extended antibiotic prophylaxis.

**Table 3:** Examining the factors affecting the development of complications with regression modeling.

		Model I			Model II			Model III		
		OR	95% CI	*p*	OR	95% CI	*p*	OR	%95CI	*p*
Extended Prophylaxis status	**Negative**	Reference			Reference			Reference		
	**Positive**	2.639	0.792–8.792	0.114	0.901	0.195–4.153	0.894	0.859	0.182–4.041	0.847
Duration of surgery (minutes)					1.018	1.006–1.031	0.004	1.018	1.005–1.031	0.005
Age(years)								1.036	0.975–1.100	0.259
Sex	**Female**	Reference			Reference			Reference		
	**Male**							0.001	0.001 *	0.999

**OR**: odds ratio, **95% CI**: 95% confidence interval for OR, **Nagelkerke R^2^**: Model I: 0.038; Model II: 0.155; Model III: 0.181. * The upper limit of the confidence interval could not be calculated because it was too far from the level of statistical significance within the model.

## Data Availability

The datasets generated during and/or analyzed during the current study are not publicly available, but are available from the corresponding author on reasonable request.
